# Supplementary material for: From sequence to enzyme mechanism using multi-label machine learning
Source: BMC Bioinformatics. 2014 May 19;15:150. doi: 10.1186/1471-2105-15-150 (PMC4229970; doi:10.1186/1471-2105-15-150)
Supplement: Additional file 2 — Java code of ml2db. Additional file ml2db_code.tar.gz contains the Java source code to run the multi-label machine learning experiments and save the results to database. The code’s Javadoc is included. [file 1471-2105-15-150-S2.zip › additional file 2/ml2db/ml2db/doc/index-files/index-16.html]

P-Index


JavaScript is disabled on your browser.


- Overview
- Package
- Class
- Use
- Tree
- Deprecated
- Index
- Help

- Prev Letter
- Next Letter

- Frames
- No Frames

- All Classes

A B C D E F G H I J K L M N O P Q R S T U V W X 


## P

path - Static variable in class test.ml2db.mulan.measuremetrics.AnnotationsReaderTest


PDF - Static variable in class main.ml2db.graphviz.GraphViz


Picker - Interface in main.ml2db.al.utility.pickers
:   A class that picks and returns an instance (random picking or ordering by
    frequency in the data etc.)

PLAIN - Static variable in class main.ml2db.graphviz.GraphViz


PLOTS\_FOLDER - Static variable in class test.ml2db.dataharness.TestProjectParameters


PNG - Static variable in class main.ml2db.graphviz.GraphViz


postProcessDistances(double[]) - Method in class main.ml2db.weka.JaccardDistanceStored


pred1 - Static variable in class test.ml2db.mulan.measuremetrics.AnnotationsReaderTest


PRED\_RECORDS\_TABLE\_NAME - Static variable in class main.ml2db.mulan.predict.PredictionsRecordTable


predict() - Method in class main.ml2db.mulan.predict.PredictionsSaver


PREDICTION\_EXPERIMENTS\_TABLE\_NAME - Static variable in class main.ml2db.mulan.predict.PredictionsExperimentTable
:   the name for the table to store the predictions experiments records

PREDICTIONS\_DATA - Static variable in class test.ml2db.dataharness.TestProjectParameters


PREDICTIONS\_FOLDER - Static variable in class test.ml2db.dataharness.TestProjectParameters


PREDICTIONS\_PATH - Static variable in class main.ml2db.ProjectParameters


PredictionsExperimentTable - Class in main.ml2db.mulan.predict


PredictionsExperimentTable(DbManager) - Constructor for class main.ml2db.mulan.predict.PredictionsExperimentTable


PredictionsPerformanceMeasurer - Class in main.ml2db.mulan.measuremetrics
:   Generates prediction metrics by comparing 1.

PredictionsPerformanceMeasurer(String, String, String, String, String) - Constructor for class main.ml2db.mulan.measuremetrics.PredictionsPerformanceMeasurer


PredictionsPerformanceMeasurerTest - Class in test.ml2db.mulan.measuremetrics


PredictionsPerformanceMeasurerTest() - Constructor for class test.ml2db.mulan.measuremetrics.PredictionsPerformanceMeasurerTest


PredictionsRecordTable - Class in main.ml2db.mulan.predict
:   The table containing the predictions (experiment identifier, instance, label,
    confidence etc.) for a prediction experiment.

PredictionsRecordTable(DbManager) - Constructor for class main.ml2db.mulan.predict.PredictionsRecordTable


PredictionsSaver - Class in main.ml2db.mulan.predict
:   A class to create and save some machine learning predictions.

PredictionsSaver(CrossExperimenter, MultiLabelInstances, MultiLabelInstances, MultiLabelLearner, boolean, boolean, int) - Constructor for class main.ml2db.mulan.predict.PredictionsSaver
:   Predictor hooked into a cross-evaluation experiment.

PredictionsSaver(DbManager, int, int, IdMultiLabelInstances, IdMultiLabelInstances, MultiLabelInstances, MultiLabelInstances, MulanLearner, MultiLabelLearner, boolean, int) - Constructor for class main.ml2db.mulan.predict.PredictionsSaver


PredictionsSaver(TrainTestExperimenter, MultiLabelInstances, MultiLabelInstances, MultiLabelLearner, boolean) - Constructor for class main.ml2db.mulan.predict.PredictionsSaver
:   Predictor hooked into a train-test evaluation experiment.

PredictionsSaverTest - Class in test.ml2db.mulan.predict
:   Before running, make sure the predictions record test is myIsam: ALTER TABLE
    `test`.`prediction\_records` ENGINE = MyISAM ; and instance field is longer:
    ALTER TABLE `test`.`prediction\_records` CHANGE COLUMN `instance` `instance`
    VARCHAR(15) NULL DEFAULT NULL ;
    Train arff: inst1, inst2 etc.

PredictionsSaverTest() - Constructor for class test.ml2db.mulan.predict.PredictionsSaverTest


predictionsTable() - Method in class main.ml2db.mulan.predict.PredictionsSaver


predictor1TrainOnTrain() - Static method in class test.ml2db.mulan.predict.PredictionsSaverTest


prepareMeasures(boolean, boolean, int) - Method in class main.ml2db.mulan.measuremetrics.PredictionsPerformanceMeasurer


printDist(Instance, Instance, double) - Static method in class main.ml2db.weka.JaccardDistanceTest


printInst(GlobalDistanceMetric, IdInstance) - Method in class test.ml2db.al.utility.globaldistance.GlobalDistanceMetricTest


printNeighbours(Instance, Instances) - Method in class main.ml2db.mulan.classifier.lazy.BRkNNVisible


printNeighboursForGraphviz(String, NeighbourhoodNetwork, boolean) - Static method in class main.ml2db.graphviz.GraphVizPrinter


printPhiCorrelations() - Method in class main.ml2db.mulan.mulanarff.Statistics
:   Prints out phi correlations

printPhiDiagram(double) - Method in class main.ml2db.mulan.mulanarff.Statistics
:   This method prints data, useful for the visualization of Phi per dataset.

priors() - Method in class main.ml2db.mulan.mulanarff.Statistics
:   returns the prior probabilities of the labels

PROJECT\_NAME - Static variable in class main.ml2db.ProjectParameters


ProjectParameters - Class in main.ml2db
:   Contains file paths (database connection files, arff properties files, mulan
    xml files etc.)

ProjectParameters() - Constructor for class main.ml2db.ProjectParameters


propertiesAreCorrect(Properties) - Method in class main.ml2db.weka.ArffProps


propertiesAreCorrect() - Method in class main.ml2db.weka.DataSetChecker


propertiesKeys() - Method in class main.ml2db.mulan.mulanarff.MulanArffProperties


propertiesKeys() - Method in class main.ml2db.weka.ArffProps
:   All properties: core + statistics

PROPS\_PATH - Static variable in class test.ml2db.dataharness.TestProjectParameters


PS - Static variable in class main.ml2db.graphviz.GraphViz

A B C D E F G H I J K L M N O P Q R S T U V W X

- Overview
- Package
- Class
- Use
- Tree
- Deprecated
- Index
- Help

- Prev Letter
- Next Letter

- Frames
- No Frames

- All Classes
